# Supplementary material for: Low-Density Lipoprotein Receptor Is a Key Driver of Aggressiveness in Thyroid Tumor Cells
Source: Int J Mol Sci. 2023 Jul 6;24(13):11153. doi: 10.3390/ijms241311153 (PMC10342163; doi:10.3390/ijms241311153)
Supplement: Supplementary file 1 [file ijms-24-11153-s001.zip › ijms-2450077-supplementary.pdf]

Table S1. List of antibodies used for Western Blot studies

Antibody	Species	Dilution	Company (catalog#)
LDLR	Rabbit	1:200	Abcam EP1553Y
p44/42 MAPK (ERK 1/2) = **total ERK**	Rabbit	1:1000	Cell signaling (#9102)
P-p44/42 MAPK MAPK (ERK 1/2) = **p-ERK**	Rabbit	1:1000	Cell signaling (#9101)
AKT	Rabbit	1:1000	Cell signaling (#4685)
p-AKT	Rabbit	1:1000	Cell signaling (#9271)
MTOR	Rabbit	1:200	Cell signaling (#2972)
p-MTOR	Rabbit	1:200	Cell signaling (#2971)
NF-κB	Rabbit	1:1000	Cell signaling (#3035)
Anti-rabbit secondary antibody		1:5000	Promega (W4011)
Anti-mouse secondary antibody		1:5000	Promega (W4021)

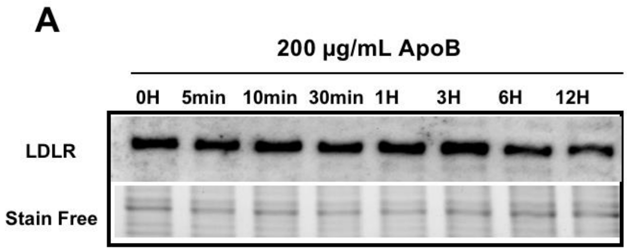

Figure S1. Western blot analysis showing the time course of Low-Density lipoprotein (LDLR) protein expression in the TPC1 cell line after low-density lipoprotein (LDL) (200 µg/mL ApoB) incubation for 5 min, 10 min, 30 min, 1 h, 3 h, 6 h and 12 h . Western blot Stain-Free gel was used as the loading control.

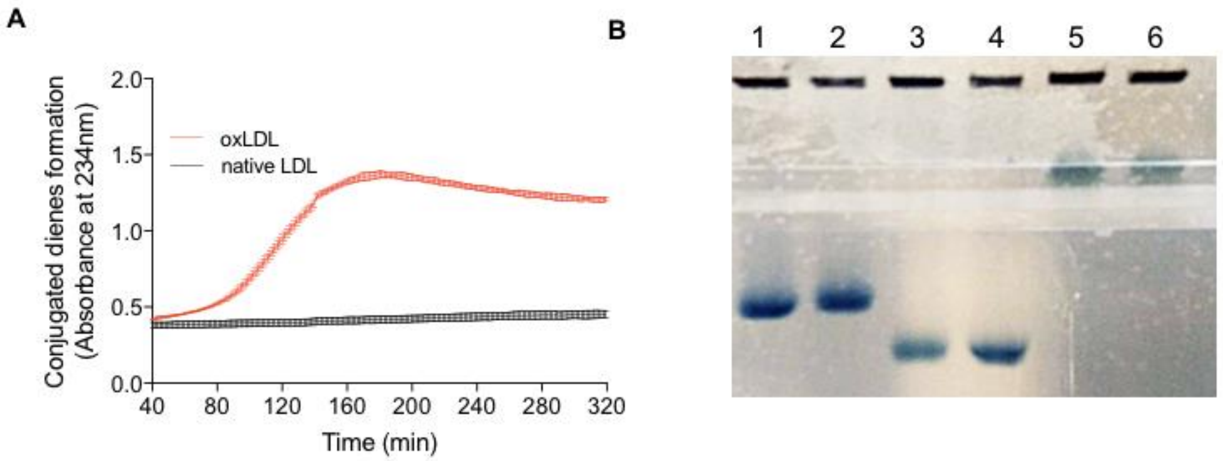

Figure S2. A) Diene formation curves of native LDL incubated with media and LDL after being under specific oxidation conditions. (B) Electrophoretic mobility of native LDL and LDL after being exposed to oxidation or partially oxidized as shown in panel A. 1 & 2: partially oxidated LDL; 3 & 4: oxidated LDL; 5 & 6: native LDL (used in our experiments).

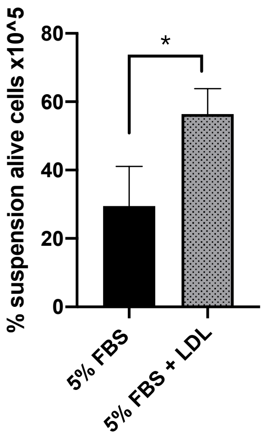

Figure S3. The graph represents the percentage of suspension alive BCPAP cells treated with LDL (200 µg/mL ApoB) compared to the suspension alive BCPAP cells at basal conditions (5% FBS). Statistical analysis: An unpaired t-test was performed to compare the LDL-treated cells with the control condition (*p = 0.0284). The results corresponded to the mean ± SEM of three independent experiments.

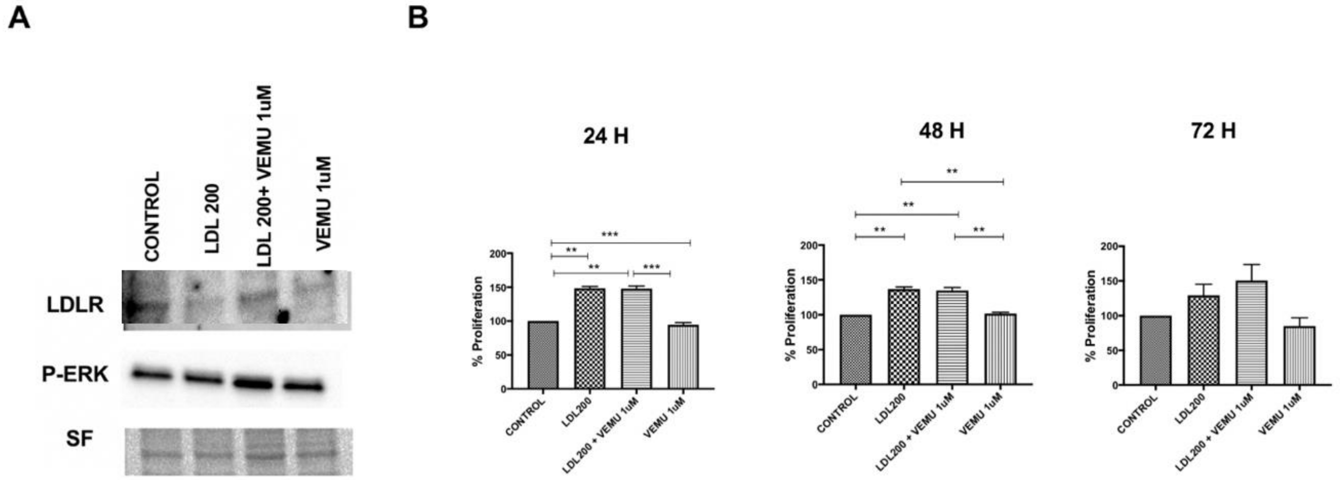

Figure S4. Western blot protein expression panel and MTT assay of TPC1 cell lines after LDL (200 µg/mL ApoB) incubation and vemurafenib treatment (1 µM). (A) Western blot protein expression panel of LDLR, and p-ERK after LDL (200 µg/mL ApoB) incubation and vemurafenib treatment (1 µM) for 24 h. Stain-Free (SF) gel was used as the loading control. (B) Proliferation percentage determined via MTT assay in the TPC1 cell line after LDL (200 µg/mL ApoB) incubation and vemurafenib treatment (1 µM) compared to the control condition (5% FBS) at 24 h, 48 h, and 72 h. Statistical analysis: One-way ANOVA test plus Tukey's multiple comparisons test (*$p < 0.01$, **$p < 0.001$, ***$p = 0.0008$). Data are expressed as mean ± SEM (n = 3) of three independent experiments carried out in quintuplicate.

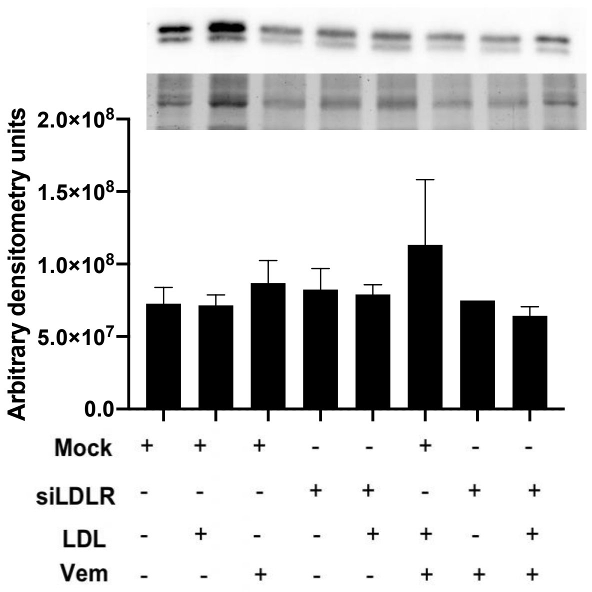

Figure S5. ERK protein expression panel in BCPAP cell line treated with LDL, siLDLR and/or vemurafenib. Representative blot is shown of ERK. Stain-Free (SF) gel was used as the loading control. Graph shows densitometry analysis of the Western Blots of ERK after siLDLR, LDL (200 µg/mL ApoB) incubation and/or vemurafenib treatment (1 µM) for 48 h in comparison to LDL-alone condition (LDL + DMSO 0,1% + Mock).

SUPPLEMENTARY MATERIAL: Cell lines and cell culture

The supplementary experiments depicted in Figure S6 were carried out on cell lines derived from anaplastic human thyroid carcinoma, CAL-62 (bearing KRAS p.G12), and 8505C (bearing the BRAF V600E oncogene). Both cell lines were provided by Paolo Vigneri of Azienda Ospedaliero Universitaria Policlinico Vittorio Emanuele Catania, Catania, Sicilia, IT. CAL-62 cells were cultured in DMEM High Glucose and 8505C cells were cultured in RPMI 1940, both mediums were supplemented with 10% FBS, 100 U/ml penicillin, and 1 µg/ml streptomycin at 37 °C in a 5% CO2 atmosphere.

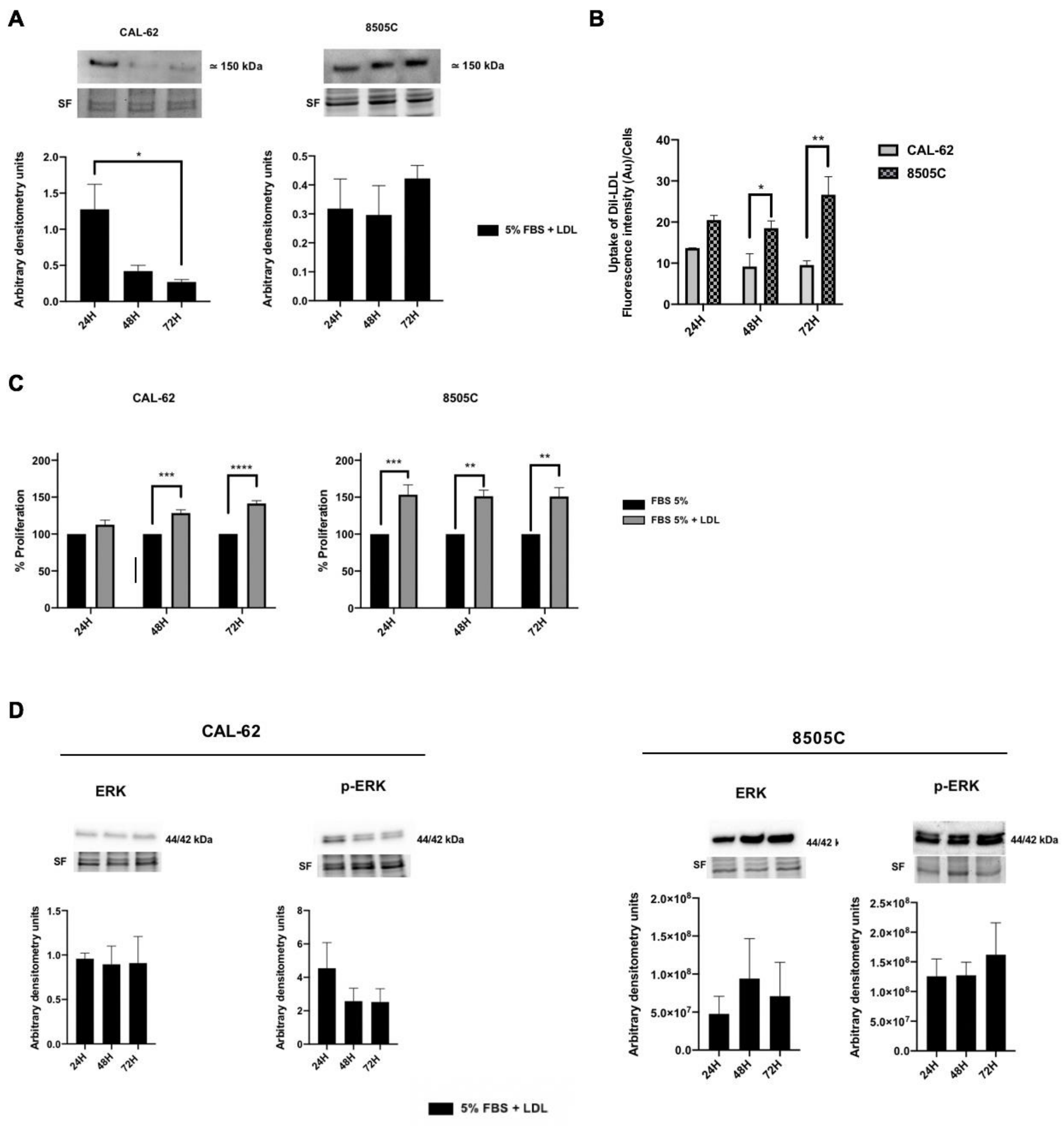

Figure S6. LDLR protein expression, 19-dioctadecyl-3,3,39,39-tetramethyl indocarbocyanine (DiI)-LDL uptake, percentage of cellular proliferation and RAS/RAF/MAPK (MEK)/ERK pathway expression in CAL-62 and 8505C cell lines. A) Cells were treated with LDL (200 µg/mL ApoB) and harvested at 24 h, 48 h, and 72 h before analysis. One representative blot is shown: Stain-Free gel (SF) was used as the loading control. Graphs show densitometry of the Western Blots relative to LDL-treated cells. Statistical test: A one-way ANOVA test plus Tukey's multiple comparisons test (*p = 0.032). Data are expressed as mean ± SEM of a minimum of three independent experiments (n = 3). (B) Cells were exposed to DiI-LDL (200 µg/mL ApoB) for 24 h, 48 h, and 72 h before analysis for mean fluorescence intensity by fluorescence spectrometer to compare LDL uptake between both cell lines. Statistical analysis: A two-way ANOVA

test plus Sidak's multiple comparisons test were performed to compare the DiI-LDL uptake between the two groups at each time point (*$p < 0.03$, **$p < 0.002$). Data are expressed as mean ± SEM of a minimum of three independent experiments (n = 3). C) Percentage of cellular proliferation determined by MTT assay of the CAL-62 and 8505C cell lines. Cells were treated with basal conditions (5% FBS), as a control, or with LDL (200 µg/mL ApoB) for 24 h, 48 h, and 72 h. Statistical analysis: A two-way ANOVA test plus Sidak's multiple comparisons test were performed to compare both groups (***$p = 0.0007$, ****$p < 0.001$). Data are expressed as mean ± SEM of three independent experiments (n = 3) carried out in quintuplicate. D) Western blot analysis for RAS/RAF/MAPK (MEK)/ERK pathways. CAL-62 and 8505C cells were treated with LDL (200 µg/mL ApoB) and harvested at 24 h, 48 h, and 72 h. Representatives blots are shown. SF gel was used as the loading control. Graphs show densitometry of the Western Blots. Statistical analysis: A one-way ANOVA test plus Tukey's multiple comparisons test. Data are expressed as mean ± SEM of a minimum of three independent experiments (n = 3).
